# Metabolomic Analysis of Plasma from *GABA_B(1)_* Knock-Out Mice Reveals Decreased Levels of Elaidic *Trans*-Fatty Acid

**DOI:** 10.3390/metabo10120484

**Published:** 2020-11-26

**Authors:** Claudia Fattuoni, Luigi Barberini, Antonio Noto, Paolo Follesa

**Affiliations:** 1 Department of Chemical and Geological Sciences, University of Cagliari, 09042 Monserrato, Italy; 2 Department of Medical Sciences and Public Health, University of Cagliari, 09042 Monserrato, Italy; barberini@unica.it (L.B.); antonionoto@unica.it (A.N.); 3 Department of Life and Environment Sciences, Section of Neuroscience and Anthropology and Center of Excellence for Neurobiology of Dependence, University of Cagliari, 09042 Monserrato, Italy; follesa@unica.it

**Keywords:** murine model, plasma, metabolomics, GC-MS, elaidic acid

## Abstract

Mice lacking the GABA_B(1)_ subunit of gamma-aminobutyric acid (GABA) type B receptors exhibit spontaneous seizures, hyperalgesia, hyperlocomotor activity, and memory impairment. Although mice lacking the GABA_B(1)_ subunit are viable, they are sterile, and to generate knockout (KO) mice, it is necessary to cross heterozygous (HZ) mice. The aim of our study was to detect the metabolic differences between the three genotypes of *GABA_B(1)_* KO mice in order to further characterize this experimental animal model. Plasma samples were collected from wild-type (WT), HZ, and KO mice. Samples were analyzed by means of a gas chromatography-mass spectrometry (GC-MS) platform. Univariate *t*-test, and partial least square discriminant analysis (PLS-DA) were performed to compare the metabolic pattern of different genotypes. The metabolomic analysis highlighted differences between the three genotypes and identified some metabolites less abundant in KO mice, namely elaidic acid and other fatty acids, and chiro-inositol.

## 1. Introduction

Gamma-aminobutyric acid (GABA) regulates inhibitory neurotransmission in the adult mammalian brain acting on GABA_A_ and GABA_B_ receptors, and both types of receptors are involved in the fine and precise regulation of neuronal excitability. GABA_B_ receptors are G protein-coupled receptors and exist as heterodimers composed by the R1 and R2 subunits encoded by different genes [1]. Accumulating evidences suggest that GABA receptors may promote cell migration, differentiation, and synaptogenesis, therefore affecting Central Nervous System (CNS) development. Accordingly, the knocking down of GABA_B_ receptors in mice, by eliminating the gene coding for the R1 subunit (*GABA_B(1)_* knock-out), causes behavioral alterations and abnormalities including epilepsy, impaired memory, hyperalgesia, hyperthermia, and hyperactivity [2,3,4,5,6]. Moreover, recent findings in our laboratory demonstrate that both *GABA_B(1)_* knockout (KO) and heterozygous (HZ) mice consumed high amounts of ethanol and prefer to drink alcohol rather than water [7] and could be used as an experimental model to study alcohol addiction.

All these observations suggest a rearrangement in the neuronal networks and altered neuronal excitability in the CNS of these mice [7] accompanied by changes in expression of a number of genes. Accordingly, *GABA_B(1)_* KO mice are characterized by altered levels of mRNA encoding for the different GABA_A_ receptor subunits [7] as well as growth factors, transcription factors, and enzymes encoding genes (Follesa et al. unpublished observations), and these alterations in gene expression may be regulated by changes in DNA methylation [8,9]. Nevertheless, little is known about the functional consequences that these changes can produce, and to the best of our knowledge, no information is available on metabolomic studies performed in *GABA_B(1)_* KO mice. To further characterize this mouse strain, the aims of our research were to investigate the metabolic differences between plasma from three genotypes of mice obtained by crossing *GABA_B(1)_* HZ mice. The ultimate goal of this study was to characterize *GABA_B(1)_* KO mice and identify metabolic routes than could be exploited to further understand the molecular, cellular, and functional alterations that could play a pivotal role in epilepsy, impaired memory, hyperalgesia, hyperthermia, hyperactivity [2,3,4,5,6], changes in gene expression, and drinking behavior [7] observed in these mice and give some new insights for intervention to treat and/or restore these CNS alterations. Biological samples, such as blood, can provide comprehensive information being minimally invasive to obtain, rendering it a good medium for clinical diagnostics.

## 2. Results

Plasma samples from 82 mice (32 WT, 41 HZ, and 9 KO) underwent metabolomic analysis. The three possible genotypes were compared in different combinations: WT vs. HZ, WT vs. KO, and HZ vs. KO.

**Figure 1:**
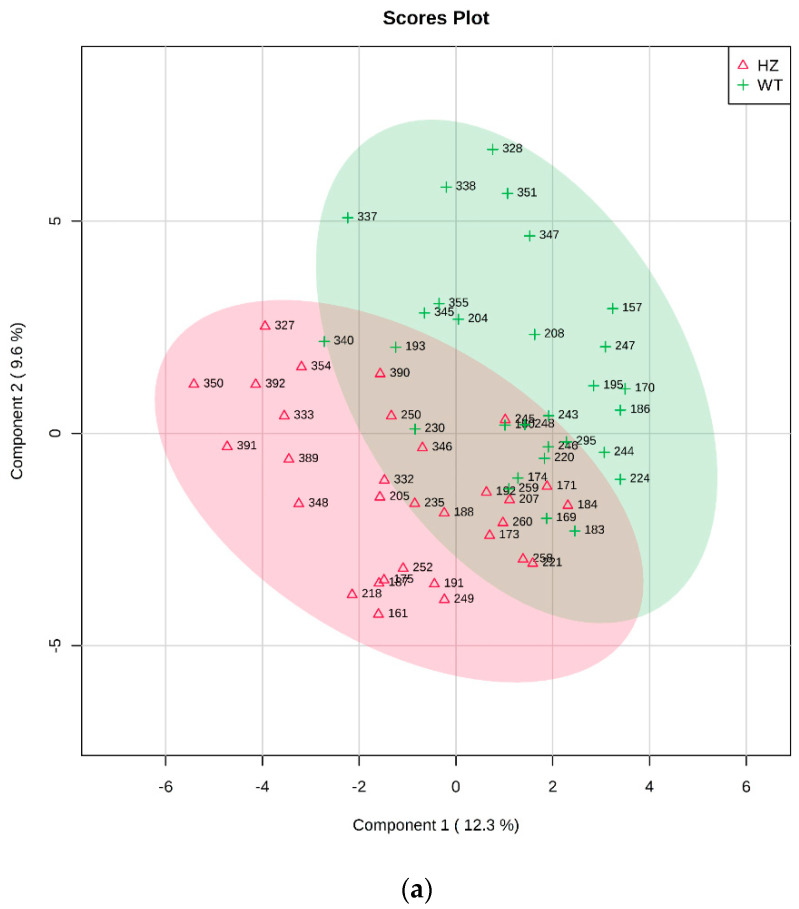

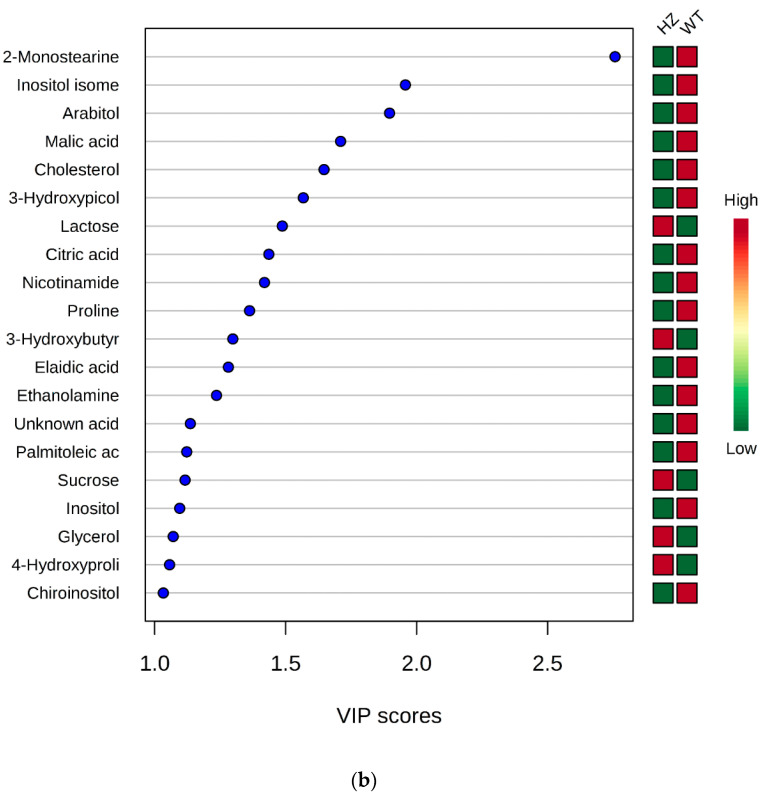
PLS-DA analysis of WT vs. HZ. (**a**) Two-dimensional (2D) score plot, and (**b**) the corresponding VIP score plot (score > 1.0).

WT vs. HZ: After a preliminary outlier removal, the groups resulted formed by 29 WT and 31 HZ. Univariate analysis did not reveal any significant metabolite. Partial least square discriminant analysis (PLS-DA) analysis allowed to build a statistically validated model (*p* < 0.01): the relative two-dimensional (2D) score plot and the corresponding VIP (Variable Importance in Projection) score were plotted and are reported in Figure 1a,b, respectively.

All metabolites resulted more abundant in WT except for lactose, 3-hydroxybutyric acid, sucrose, glycerol, and 4-hydroxyproline (Figure 1b).

WT vs. KO: Two groups formed by 29 (WT) and 9 (KO) were submitted to statistical analysis. The *t*-test revealed the elaidic acid as statistically different between groups (*p* = 4.1417 × 10^−4^, FDR (False Discovery Rate) = 0.0207). The unequivocal identification of elaidic acid, in comparison with the more abundant *cis*-isomer oleic acid, is reported as Supplementary Figures S1–S3.

**Figure 2:**
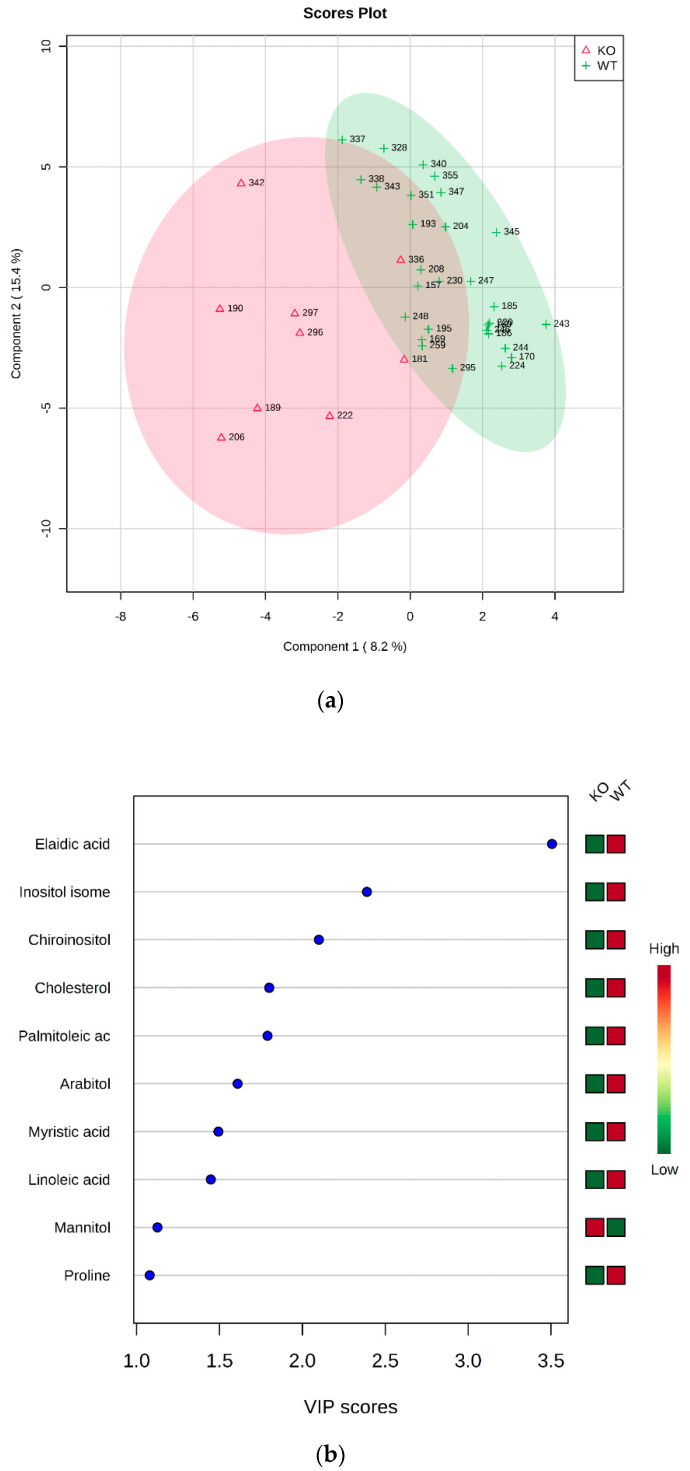
PLS-DA analysis of WT vs. KO. (**a**) 2D scores plot, and (**b**) the corresponding VIP score plot (score > 1.0).

The PLS-DA analysis allowed to build the model whose plots are reported in Figure 2. As evident from the VIP score plot, all metabolites resulted more abundant in WT, except for mannitol (Figure 2b).

**Figure 3:**
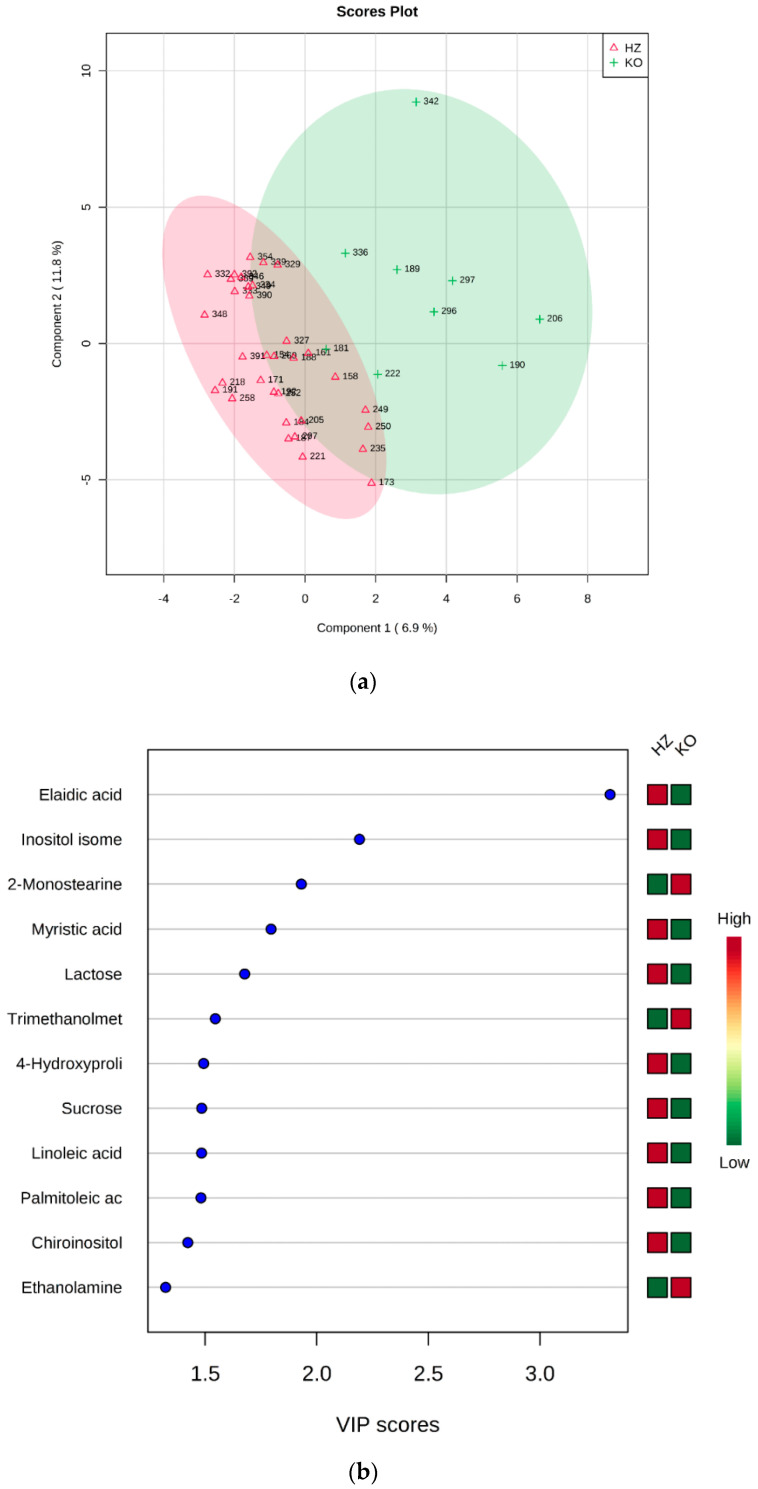
PLS-DA analysis of HZ vs. KO. (**a**) 2D scores plot, and (**b**) the corresponding VIP score plot (score > 1.0).

HZ vs. KO: Two groups formed by 34 (HZ) and 9 (KO) were submitted to statistical analysis. Again, the *t*-test revealed the elaidic acid as statistically different between groups (*p* = 6.9305 × 10^−4^, FDR = 0.034652). The PLS-DA analysis allowed to build the model whose plots are reported in Figure 3.

The statistical parameters of the three PLS-DA models are reported in Table 1, and the corresponding metabolite variations are summarized in Table 2.

**Figure 4:**
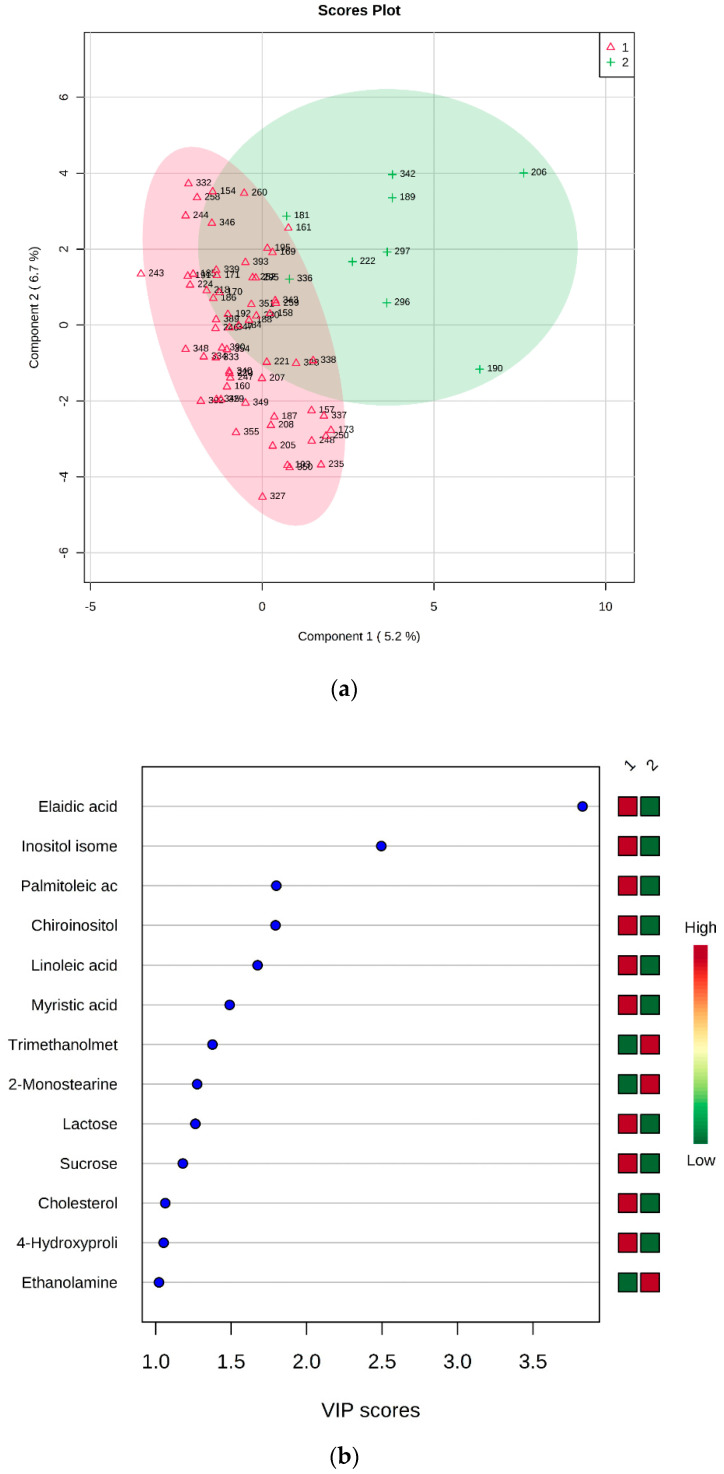
PLS-DA analysis of WT + HZ (group 1) vs KO (group 2). (**a**) 2D scores plot, and (**b**) the corresponding VIP score plot (score > 1.0).

Fatty acids, namely elaidic, linoleic, myristic, and palmitoleic acids, appeared to be less abundant in KO mice in comparison with both WT and HZ. Analogous behavior was shown by chiro-inositol and an undefined inositol isomer (other than myo- and scyllo-inositol). Due to the apparent similarity of WT and HZ, the combined group WT + HZ was compared with KO (69 vs. 9 samples), obtaining the PLS-DA model reported in Figure 4. This model was best described by the first three components, and the cross validation provided accuracy = 0.88732, R^2^ = 0.6844, Q^2^ = 0.23364. The permutation test on 100 permutations gave *p* = 0.4.

As expected, all metabolites resulted more abundant in the group formed by WT and HZ, except for trimethanolmethylamine, 2-monostearine, and ethanolamine (Figure 4b).

Elaidic acid resulted as the statistically most significant metabolite in all comparisons involving KO mice, being found in lower amounts in plasma when compared either with HZ or WT. Elaidic acid, the *trans* isomer of oleic acid, mainly derives from diet, being a component of ruminant fat, meat, and dairy products. To investigate the source of elaidic acid in our samples, the mice food was analyzed. The GC-MS analysis of the lipophilic phase revealed a fatty acid composition in agreement with what was declared by the food manufacturer, with linoleic, oleic, and palmitic acid being the most abundant. We found a small amount of elaidic acid, around 1% of total fatty acid content, most likely derived from the whey powder declared by the manufacturer in the food formula.

## 3. Discussion

The comparison of the metabolite content of plasma samples from different genotype mice revealed several interesting features in the animals lacking GABA_B_ receptors. KO mice were characterized by lower amounts of fatty acids when compared with HZ or WT (alone or grouped together). In particular, the most important fatty acids were elaidic, linoleic, myristic, and palmitoleic. The greatest impact can be ascribed to elaidic acid, its different concentration between KO and either WT or HZ is clearly revealed also by univariate (*t*-test) analysis. Elaidic acid is the *trans*-isomer of C18 mono-unsaturated oleic acid, its occurrence in biologic samples is mainly ascribed to exogenous sources. Accordingly, elaidic acid is usually found in ruminant-derived food [10], and is degraded by β-oxidation in mitocondria, as reported by Yu [11], although with a less efficient mechanism than saturated or *cis*-unsaturated fatty acids. Fluctuation in the concentrations of these compounds may affect cellular functions that could be involved in the pathophysiology and behavioral phenotype observed in these KO mice. It has been reported that elaidic acid supplementation on pregnant mice induced a pro-inflammatory and adipogenic transcriptional profile compared with the oleic *cis*-isomer, leading to global adipose tissue DNA hypermethylation in the progeny [8]. Moreover, elaidic acid and linolelaidic acid enhanced extracellular ATP-induced apoptosis, with elevated activation of the apoptosis signal-regulating kinase 1 ASK1 pathway in a macrophage-like cell line. *Trans* fatty acids promote extracellular ATP-induced apoptosis by targeting ASK1 and suggest new TFA-associated pathways leading to inflammatory signal transduction and cell death leading to onset and progression of atherosclerosis [12]. We cannot rule out the possibility that *trans* fatty acids may affect apoptosis also in other cell types, including neurons, and that the reduction of *trans* fatty acid concentrations we observed in *GABA_B(1)_* KO mice could play a role in regulating the increased hippocampal neurogenesis measured in *GABA_B(1)_* KO mice (Follesa et al., unpublished observation) by reducing hippocampal apoptosis. These alterations in hippocampal cell proliferation might be part of a neuronal circuitry rearrangement in the CNS of *GABA_B(1)_* KO mice that involves also the gene expression of GABA_A_ receptors and related behavior [7] and elaidic acid may alter the function of GABA_A_ receptors. Accordingly, it was found that a mixture of eight fatty acids, comprising elaidic acid, produce anxiolytic-like effects comparable to diazepam in Wistar rats, exerting these effects through GABA_A_ receptors [13]. In agreement with these observations, *GABA_B(1)_* KO mice showed an anxiety behavior [14] as well as depressive [15,16] and the lower levels of elaidic acid we observed may exacerbate these condition in *GABA_B(1)_* KO mice.

The lower amounts of fatty acids in KO mice could be ascribed to a higher degradation in *GABA_B(1)_* KO mice as these mice have altered metabolic activity. These metabolic alterations may be necessary to counteract the higher energy expenditure [17]. In our investigation, we did not measure food intake, but we did not observe significant changes in body weight between the three genotypes. Other studies demonstrated that *GABA_B(1)_* KO mice consume about 15% more food compared to control WT mice and this was accompanied by a strong reduction in plasma leptin levels [18], but at the same time, they show a 50% reduction of white adipose tissue (WAT), and increase in the weight of organs such as heart and brain [18]. This higher energy expenditure need may be the result of hyperlocomotor activity, wild running, as well as frequent spontaneous seizures. Accordingly, *GABA_B(1)_* KO mice are characterized by spontaneous seizures, hyperalgesia, hyperlocomotor [2,3,4,5,6] activity, and memory impairment [19]. On the other hand *trans* fatty acids may act on DNA methylation that could regulate the changes in gene expression observed in these mice [7]. All these allostatic adaptive mechanisms, adopted to survive the lack of GABA_B_ receptors, may also be responsible for the large variability observed in some of the parameters we measured in these germinal knock-out mice.

Two other metabolites found in lower amounts in KO mice (and also in HZ when compared with WT) were chiro-inositol and a not precisely identified inositol isomer (other than myo- and scyllo-inositol). Chiro-inositol is one of the nine stereoisomers of 1,2,3,4,5,6-hexahydroxycyclohexane, and myo-inositol is the most commonly found in nature. Chiro-inositol in vivo is converted in myo-inositol [20], and is a component of an inositolphosphoglycan insulin mediator found in rat liver, stimulating pyruvate dehydrogenase phosphatase, kinetically similar to insulin. Its diet supplementation was found to increase the effect of insulin in patients with the polycystic ovary syndrome, improving ovulatory function and decreasing serum androgen concentrations, blood pressure, and plasma triglyceride concentrations [21]. In view of the above considerations, we cannot exclude that *GABA_B(1)_* KO mice could have a dysregulation in insulin and insulin-mediated pathways that needs to be further investigated in future studies.

The limitation of this study essentially lies in the low number of samples, especially for the KO group. This is a pilot study and the data reported, although very preliminary, allow to extract some interesting data leading the way to supplementary studies on bigger cohorts.

## 4. Materials and Methods

All procedures were performed in accordance with the European legislation EU Directive 2010/63, the “Guide for Care and Use of Laboratory Animals” adopted by NIH, USA (8th edition, 2011) and were approved by the Animal Ethics Committee of the University of Cagliari and by Italian Ministry of Health (D.M. 39/94-A 25-03-1994, Communication UNICA 29.11.2010, D.M. n° 128/2012-A 04.06.2012 and authorization UNICA #1-03/2015). Animals were housed in groups of three to six in standard conditions of temperature (21 ± 1 °C) and humidity (60%) under a 12 h light/dark cycle (lights on at 7:00 A.M.) with food and water available ad libitum. All efforts to minimize animal discomfort and to reduce the number of animals used were made.

### 4.1. Animals and Sample Study Population

Balb/c *GABA_B(1)_* germinal KO mice were generously provided by Dr. Martin Gassmann (University of Basel, Switzerland) and have been previously described [19]. In order to prevent genetic drift, after about 20 generations (F20), the Balb/c HZ mice were crossed with WT mice of the FVB (Friend Virus B NIH Jackson) strain to obtain new HZ mice (F1). The F1 HZ mice where then crossed again to keep the colony until the next F20. Therefore, the strain of animal used in our laboratories is different from the original [7] due to the crossing with the FVB strain. Over the years, our colony of mice was maintained by crossing HZ mice since *GABA_B(R1)_* knock-out mice are sterile and therefore, to obtain KO mice, we need to cross HZ ± *GABA_B(R1)_* mice [22]. The mice used in the present study derived from independent mating of the F12–F16 generation. Litters were genotyped by polymerase chain reaction (PCR) using specific primers as described previously [7,23] and only males were selected to be utilized at 8 to 10 weeks of age with a mean ± SEM (standard error mean) of 9.474 ± 0.1254 weeks with a range from 8 to 10.71 weeks, and body weights ranging from 21 to 31 g, and the means ± SEM for the different genotypes were WT 26.75 ± 0.50 (*n* = 32), HZ 25.75 ± 0.41 (*n* = 41), and KO 26.56 ± 0.91 (*n* = 9). In agreement with other studies [2,18], we did not observe any statistical differences in body weight between the three different genotypes (One-way analysis of variance (ANOVA), *p*-value = 0.2872).

### 4.2. Samples Preparation and GC-MS Analysis

A total number of 82 mice were used: 32 WT, 41 HZ, and 9 KO. Blood was collected from the trunk into heparinized tubes and centrifuged at 900× *g* for 10 min at 4 °C, as previously described [24]. The resulting plasma supernatant was frozen at −80 °C until GC-MS analysis. Plasma samples were analyzed as previously reported for human samples [25]. The method required slight adjustments for the low amount of plasma available. In brief, 50 μL of plasma were deproteinized with cold methanol (200 μL) and centrifuged for 10 min at 16,900× *g*. The upper phase (150 μL) was transferred in glass vials (1.5 mL) with glass insert (200 μL) and evaporated to dryness. Methoxyamine hydrochloride (30 μL, 0.24 M solution in pyridine) was added to each sample and left for 17 h at room temperature. MSTFA (*N*-Methyl-*N*-trimethylsilyltrifluoroacetamide, 30 μL) was added and reacted for 1 h at room temperature. The analytical platform was an Agilent 5977B GC/MS interfaced to the GC 7890B (Agilent Technologies, Palo Alto, CA, USA), with a DB-5 ms column (Agilent J&W Scientific, Folsom, CA, USA). Instrumental parameters were previously reported [25]. One μL of each sample was injected in split mode (1:2), sample depth 6 mm. Mass spectra were acquired in full scan mode using 2.28 scans/s with a mass range of 50–700 Amu. Each chromatogram was analyzed by the free software AMDIS (Automated Mass spectral Deconvolution and Identification System) (http://chemdata.nist.gov/mass-spc/amdis), allowing the automatic identification of each chromatographic peak by comparison of the relative mass spectra and retention times with those stored in an in-house library of 255 metabolites. Other metabolites, not included in the in-house library, were identified using NIST14 (National Institute of Standards and Technology’s mass spectral database) and the Golm Metabolome Database (GMD) (http://gmd.mpimp-golm.mpg.de/). In this way, 67 compounds were detected: following the identification levels defined by the Metabolomics Standards Initiative (MSI) [26], 53 were “confidently identified compounds” (level 1), 3 “putatively annotated compounds” (level 2), 9 “putatively annotated compound class” (level 3), and 2 “unknown compounds”. The Excel data sheet obtained (67 metabolites × 82 samples) was then submitted to statistical analysis.

### 4.3. Statistical Analysis

The data matrix was processed with the web-based tool MetaboAnalyst 4.0 (http://www.metaboanalyst.ca/) [27]. Missing values were replaced with the half of the minimum positive values in the original data, data were normalized by sum, log transformed, and Pareto scaled. Univariate analysis (*t*-test), principal component analysis (PCA), partial least square discriminant analysis (PLS-DA), and its associated variable importance in projection (VIP) score were obtained. PCA allowed outlier detection: outlier samples were removed before building the PLS-DA models. PLS-DA models were tested with the leave-one-out cross validation (LOOCV) method for the evaluation of statistical parameters (accuracy, correlation coefficient R^2^, cross validation coefficient Q^2^), determining the optimal number of components for each model description. Due to the tendency of PLS-DA to over fit data, all models were validated in order to understand whether the separation is statistically significant using the B/W permutation test [28]. Statistical differences in body weight by one-way ANOVA followed by Dunnett’s multiple comparisons test were performed using GraphPad Prism version 8.0.0 for Windows, GraphPad Software, San Diego, California USA, www.graphpad.com.

### 4.4. Mice Food Analysis

A piece of food (0.45 g) was ground in a glass mortar and stirred in a round bottom flask with water (10 mL), chloroform (10 mL), and methanol (10 mL) for 2 h. The two phases were separated in a separatory funnel (upper layer hydrophilic phase, lower layer lipophilic phase), dried, and derivatized. The hydro-phase was methoxymated/silylated and the lipo-phase was methylated, and both were analyzed by GC-MS, as previously reported [29].

## 5. Conclusions

Plasma samples from *GABA_B(1)_* KO mice were compared with HZ and WT from a metabolomic point of view. The KO phenotype resulted characterized by lower amounts of plasmatic fatty acids, the most important being elaidic acid, and two inositol isomers. The different metabolite content may regulate cellular and molecular modifications that could be related with some of the peculiar behaviors of KO mice, mainly related to CNS impairment.

## Figures and Tables

**Table 1:** PLS-DA parameters for the comparison between different groups.

Groups	Components	Accuracy	R^2^	Q^2^	B/W
WT/HZ (29/31)	5	0.78333	0.81112	0.25494	*p* < 0.01
WT/KO (29/9)	4	0.81579	0.83169	0.25824	*p* = 0.6
HZ/KO (34/9)	4	0.90698	0.84588	0.34994	*p* = 0.1

B/W: Between sum of the squares/Within sum of squares.

**Table 2:** PLS-DA most important metabolites (VIP = Variable Importance in the Projection; VIP score > 1) and the relative abundance differences. ↑ more abundant in the first class compared with the second; ↓ less abundant in the first class compared with the second.

Metabolite	Chemical Class	WT vs. HZ	WT vs. KO	HZ vs. KO
2-Monostearin	Lipid	↑		↓
3-Hydroxybutyric acid	Hydroxy acid	↓		
3-Hydroxypicolinic acid	Hydroxy acid	↑		
4-Hydroxyproline	Amino acid	↓		↑
Arabitol	Polyol	↑	↑	
Chiroinositol	Polyol	↑	↑	↑
Cholesterol	Steroid	↑	↑	
Citric acid	Carboxylic acid	↑		
Elaidic acid	Fatty acid	↑	↑	↑
Ethanolamine	Amine	↑		↓
Glycerol	Polyol	↓		
Inositol	Polyol	↑		
Inositol isomer	Polyol	↑	↑	↑
Lactose	Sugar	↓		↑
Linoleic acid	Fatty acid		↑	↑
Malic acid	Hydroxy acid	↑		
Mannitol	Polyol		↓	
Myristic acid	Fatty acid		↑	↑
Nicotinamide	Amide	↑		
Palmitoleic acid	Fatty acid	↑	↑	↑
Proline	Amino acid	↑	↑	
Sucrose	Sugar	↓		↑
Trimethanolmethylamine	Amine			↓
Unknown acid	Unknown	↑		

## Supplementary Materials

The following are available online at https://www.mdpi.com/2218-1989/10/12/484/s1, Figure S1: GC-MS chromatograms of pure standards of trimethylsilyl esters of oleic and elaidic acid, Figure S2: Mass spectrum of oleic acid trimethylsilyl ester: retention time 16.927 min., Figure S3: Mass spectrum of elaidic acid trimethylsilyl ester: retention time 16.984 min.

## References

[1] Bettler B., Kaupmann K., Mosbacher J., Gassmann M. (2004). Molecular Structure and Physiological Functions of GABA_B_ Receptors. Physiol. Rev..

[2] Quéva C., Bremner-Danielsen M., Edlund A., Jonas Ekstrand A., Elg S., Erickson S., Johansson T., Lehmann A., Mattsson J.P. (2003). Effects of GABA agonists on body temperature regulation in *GABA_B(1)_*^−/−^ mice. Br. J. Pharmacol..

[3] Prosser H.M., Gill C.H., Hirst W.D., Grau E., Robbins M., Calver A., Soffin E.M., Farmer C.E., Lanneau C., Gray J. (2001). Epileptogenesis and Enhanced Prepulse Inhibition in GABAB1-Deficient Mice. Mol. Cell. Neurosci..

[4] Haller C., Casanova E., Müller M., Vacher C.-M., Vigot R., Doll T., Barbieri S., Gassmann M., Bettler B. (2004). Floxed allele for conditional inactivation of the GABA_B(1)_ gene. Genesis.

[5] Fritzius T., Bettler B. (2020). The organizing principle of GABA _B_ receptor complexes: Physiological and pharmacological implications. Basic Clin. Pharm. Toxicol..

[6] Wu Y., Chan K.F.Y., Eubanks J.H., Guin Ting Wong C., Cortez M.A., Shen L., Che Liu C., Perez Velazquez J., Tian Wang Y., Jia Z. (2007). Transgenic mice over-expressing GABA(B)R1a receptors acquire an atypical absence epilepsy-like phenotype. Neurobiol. Dis..

[7] Floris G., Cappai A.L., Asuni G.P., Isola D., Deriu S., Tocco G., Ibba A., Follesa P. (2013). Voluntary Ethanol Drinking in GABAB Knock-Out Mice and Gene Expression of GABAA Receptors. Alcohol. Clin. Exp. Res..

[8] Flores-Sierra J., Arredondo-Guerrero M., Cervantes-Paz B., Rodríguez-Ríos D., Alvarado-Caudillo Y., Nielsen F.C., Wrobel K., Wrobel K., Zaina S., Lund G. (2016). The trans fatty acid elaidate affects the global DNA methylation profile of cultured cells and in vivo. Lipids Health Dis..

[9] González-Becerra K., Ramos-Lopez O., Barrón-Cabrera E., Riezu-Boj J.I., Milagro F.I., Martínez-López E., Martínez J.A. (2019). Fatty acids, epigenetic mechanisms and chronic diseases: A systematic review. Lipids Health Dis..

[10] Stillwell W. (2016). Membranes and Human Health. An Introduction to Biological Membranes.

[11] Yu W., Liang X., Ensenauer R.E., Vockley J., Sweetman L., Schulz H. (2004). Leaky β-Oxidation of a *trans*-Fatty Acid. J. Biol. Chem..

[12] Hirata Y., Takahashi M., Kudoh Y., Kano K., Kawana H., Makide K., Shinoda Y., Yabuki Y., Fukunaga K., Aoki J. (2017). trans-Fatty acids promote proinflammatory signaling and cell death by stimulating the apoptosis signal-regulating kinase 1 (ASK1)-p38 pathway. J. Biol. Chem..

[13] Rodríguez-Landa J.F., García-Ríos R.I., Cueto-Escobedo J., Bernal-Morales B., Contreras C.M. (2013). Participation of GABAA chloride channels in the anxiolytic-like effects of a fatty acid mixture. BioMed Res. Int..

[14] Mombereau C., Kaupmann K., Froestl W., Sansig G., van der Putten H., Cryan J.F. (2004). Genetic and Pharmacological Evidence of a Role for GABA B Receptors in the Modulation of Anxiety- and Antidepressant-Like Behavior. Neuropsychopharmacology.

[15] Cryan J.F., Kaupmann K. (2005). Don’t worry ‘B’ happy!: A role for GABAB receptors in anxiety and depression. Trends Pharmacol. Sci..

[16] Mombereau C., Kaupmann K., Gassmann M., Bettler B., van der Putten H., Cryan J.F. (2005). Altered anxiety and depression-related behaviour in mice lacking GABAB(2) receptor subunits. NeuroReport.

[17] Bonaventura M.M., Catalano P.N., Chamson-Reig A., Arany E., Hill D., Bettler B., Saravia F., Libertun C., Lux-Lantos V.A. (2008). GABA _B_ receptors and glucose homeostasis: Evaluation in GABA _B_ receptor knockout mice. Am. J. Physiol. Endocrinol. Metab..

[18] Nakamura Y., Hinoi E., Takarada T., Takahata Y., Yamamoto T., Fujita H., Takada S., Hashizume S., Yoneda Y. (2011). Positive Regulation by GABABR1 Subunit of Leptin Expression through Gene Transactivation in Adipocytes. PLoS ONE.

[19] Schuler V., Lüscher C., Blanchet C., Klix N., Sansig G., Klebs K., Schmutz M., Heid J., Gentry C., Urban L. (2001). Epilepsy, Hyperalgesia, Impaired Memory, and Loss of Pre- and Postsynaptic GABAB Responses in Mice Lacking GABAB(1). Neuron.

[20] Pak Y., Huang L.C., Lilley K.J., LarnerS J. (1992). In Vivo Conversion of [3H]Myoinositolto [3H]Chiroinositol Rat Tissues. J. Biol. Chem..

[21] Nestler J.E., Jakubowicz D.J., Reamer P., Gunn R.D., Allan G. (1999). Ovulatory and Metabolic Effects of d-Chiro-Inositol in the Polycystic Ovary Syndrome. N. Engl. J. Med..

[22] Bizzozzero-Hiriart M., Giorgio N.P.D., Libertun C., Lux-Lantos V. (2020). GABAergic input through GABAB receptors is necessary during a perinatal window to shape gene expression of factors critical to reproduction such as Kiss1. Am. J. Physiol. Endocrinol. Metab..

[23] Fukui M., Nakamichi N., Yoneyama M., Ozawa S., Fujimori S., Takahata Y., Nakamura N., Taniura H., Yoneda Y. (2008). Modulation of cellular proliferation and differentiation through GABAB receptors expressed by undifferentiated neural progenitor cells isolated from fetal mouse brain. J. Cell. Physiol..

[24] Follesa P., Floris G., Asuni G.P., Ibba A., Tocco M.G., Zicca L., Mercante B., Deriu F., Gorini G. (2015). Chronic Intermittent Ethanol Regulates Hippocampal GABA(A) Receptor Delta Subunit Gene Expression. Front. Cell. Neurosci..

[25] Barberini L., Noto A., Fattuoni C., Satta G., Zucca M., Cabras M.G., Mura E., Cocco P. (2019). The Metabolomic Profile of Lymphoma Subtypes: A Pilot Study. Molecules.

[26] Sumner L.W., Amberg A., Barrett D., Beale M.H., Beger R., Daykin C.A., Fan T.W.-M., Fiehn O., Goodacre R., Griffin J.L. (2007). Proposed minimum reporting standards for chemical analysis Chemical Analysis Working Group (CAWG) Metabolomics Standards Initiative (MSI). Metabolomics.

[27] Chong J., Soufan O., Li C., Caraus I., Li S., Bourque G., Wishart D.S., Xia J. (2018). MetaboAnalyst 4.0: Towards more transparent and integrative metabolomics analysis. Nucleic Acids Res..

[28] Barberini L., Noto A., Saba L., Palmas F., Fanos V., Dessì A., Zavattoni M., Fattuoni C., Mussap M. (2016). Multivariate data validation for investigating primary HCMV infection in pregnancy. Data Brief.

[29] Fattuoni C., Mandò C., Palmas F., Anelli G.M., Novielli C., Parejo Laudicina E., Savasi V.M., Barberini L., Dessì A., Pintus R. (2018). Preliminary metabolomics analysis of placenta in maternal obesity. Placenta.

