# Supplementary material for: Metabolomic Analysis of Plasma from GABAB(1) Knock-Out Mice Reveals Decreased Levels of Elaidic Trans-Fatty Acid
Source: Metabolites. 2020 Nov 26;10(12):484. doi: 10.3390/metabo10120484 (PMC7760308; doi:10.3390/metabo10120484)
Supplement: Supplementary file 1 [file metabolites-10-00484-s001.pdf]

Supplementary Materials

Metabolomic analysis of plasma from GABAB(1) knock-out mice reveals elaidic *trans*-fatty acid impact.

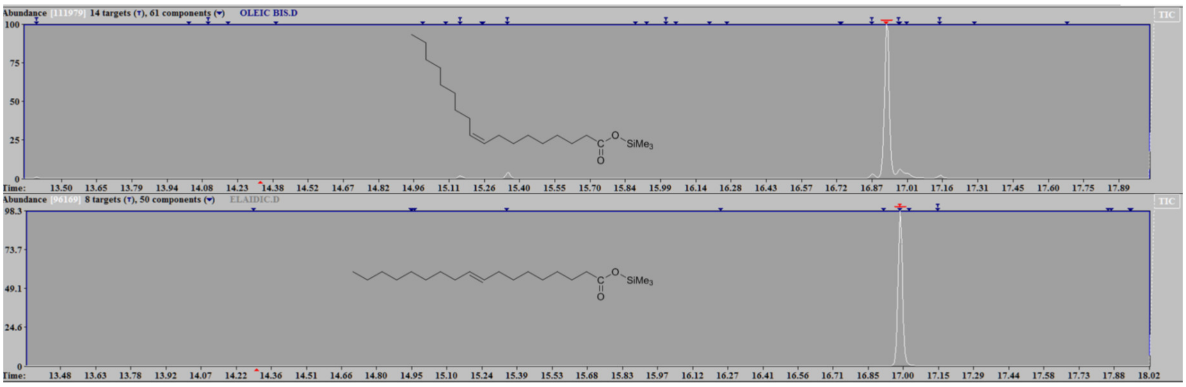

Figure S1. GC-MS chromatograms of pure standards of trimethylsilyl esters of oleic and elaidic acid.

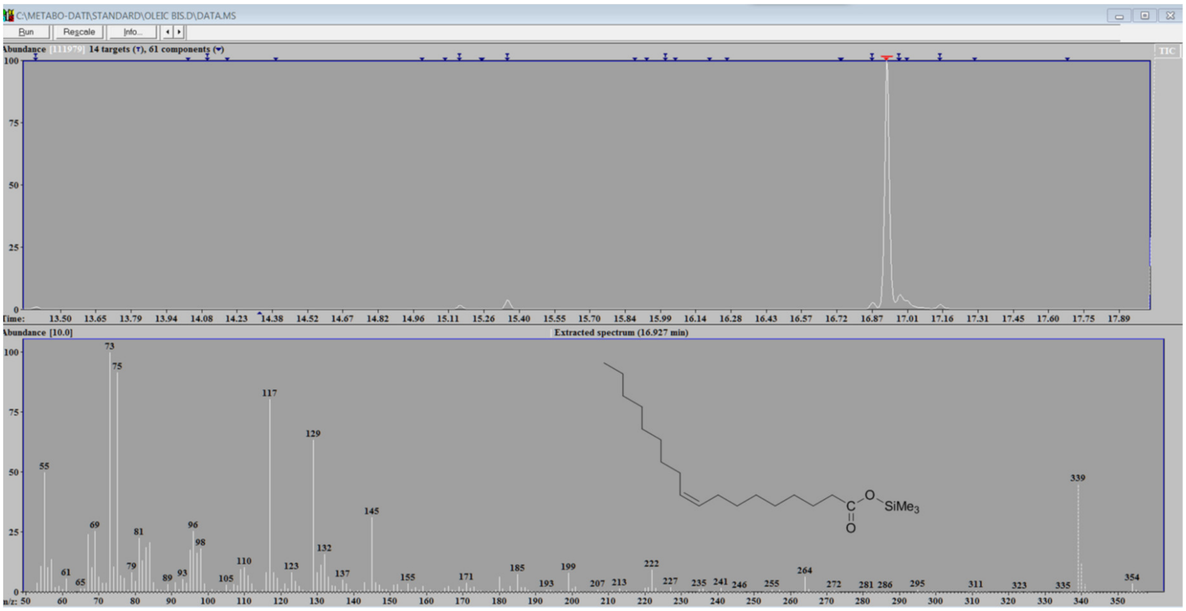

Figure S2. Mass spectrum of oleic acid trimethylsilyl ester: retention time 16.927 min.

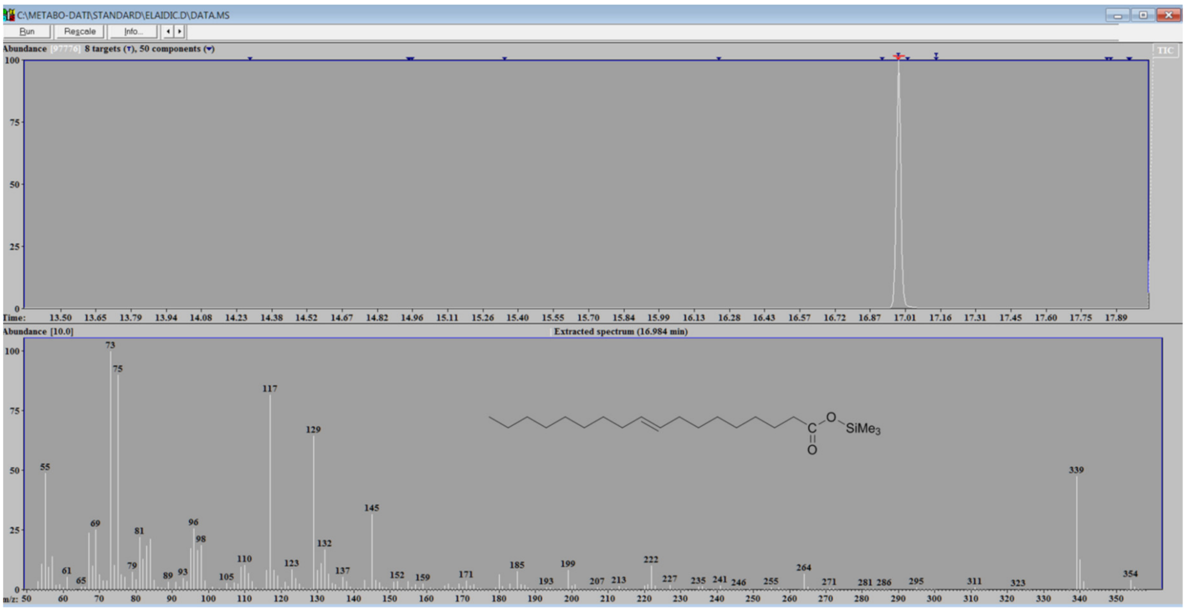

Figure S3. Mass spectrum of elaidic acid trimethylsilyl ester: retention time 16.984 min.
